# Vibration Signal Denoising Method Based on ICFO-SVMD and Improved Wavelet Thresholding

**DOI:** 10.3390/s26020750

**Published:** 2026-01-22

**Authors:** Yanping Cui, Xiaoxu He, Zhe Wu, Qiang Zhang, Yachao Cao

**Affiliations:** 1 School of Mechanical Engineering, Hebei University of Science and Technology, Shijiazhuang 050018, China; cuiyp@hebust.edu.cn (Y.C.); 18000310722@163.com (X.H.); yachao.cao@hebust.edu.cn (Y.C.); 2 Key Laboratory of Vehicle Transmission, China North Vehicle Research Institute, Beijing 100072, China; giangzh36@gmail.com

**Keywords:** improved cordyceps fungus optimization algorithm (ICFO), successive variational mode decomposition, improved wavelet thresholding, vibration signals, denoising

## Abstract

**Highlights:**

**What are the main findings?**

- A joint denoising framework (ICFO–SVMD–improved wavelet thresholding) is proposed which adaptively optimizes SVMD parameters and sub-band thresholds for nonlinear, non-stationary vibration signals.
- Simulation and experimental results on bearing and gearbox vibration data show that the proposed method outperforms VMD, SVMD, VMD–WTD, CFO–SVMD, and traditional wavelet denoising in terms of SNR, RMSE, RVR, and SER while better preserving transient fault features.

**What are the implications of the main findings?**

- The proposed method provides a robust preprocessing tool for vibration-based condition monitoring under strong noise, improving the reliability of fault feature extraction and fault diagnosis in rotating machinery.
- The ICFO–SVMD–WTD framework is generic and can be extended to other noisy engineering signals that require joint decomposition and adaptive thresholding.

**Abstract:**

Non-stationary, multi-component vibration signals in rotating machinery are easily contaminated by strong background noise, which masks weak fault features and degrades diagnostic reliability. This paper proposes a joint denoising method that combines an improved cordyceps fungus optimization algorithm (ICFO), successive variational mode decomposition (SVMD), and an improved wavelet thresholding scheme. ICFO, enhanced by Chebyshev chaotic initialization, a longitudinal–transverse crossover fusion mutation operator, and a thinking innovation strategy, is used to adaptively optimize the SVMD penalty factor and number of modes. The optimized SVMD decomposes the noisy signal into intrinsic mode functions, which are classified into effective and noise-dominated components via the Pearson correlation coefficient. An improved wavelet threshold function, whose threshold is modulated by the sub-band signal-to-noise ratio, is then applied to the effective components, and the denoised signal is reconstructed. Simulation experiments on nonlinear, non-stationary signals with different noise levels (SNR = 1–20 dB) show that the proposed method consistently achieves the highest SNR and lowest RMSE compared to VMD, SVMD, VMD–WTD, CFO–SVMD, and WTD. Tests on CWRU bearing data and gearbox vibration signals with added −2 dB Gaussian white noise further confirm that the method yields the lowest residual variance ratio and highest signal energy ratio while preserving key fault characteristic frequencies.

## 1. Introduction

Vibration-based condition monitoring is widely used for health assessment and fault diagnosis of rotating machinery. In practical industrial environments, equipment often operates under time-varying loads, speeds, and ambient conditions, leading to vibration responses that exhibit strong nonlinearity and non-stationarity, often heavily contaminated by background noise. As a result, weak transient fault features can easily be submerged in noise, especially in the presence of compound faults, which can lead to misdiagnosis or missed diagnoses [1,2,3]. In particular, compound bearing faults generate overlapping spectral components, making it difficult for traditional diagnostic methods to effectively separate and identify individual fault modes under noisy conditions [4].

To address these challenges, various time-frequency analysis and signal decomposition techniques have been developed. Wavelet transform and its multi-resolution methods are widely employed due to their excellent time-frequency localization capabilities and ability to characterize transient impacts caused by local defects. Guo et al. combined wavelet scattering transform with an improved soft-threshold denoising algorithm to enhance compound fault features in bearings [5]. Wang et al., addressing the susceptibility of rotating machinery diagnosis to interference in noisy environments, proposed a self-learning anti-noise paradigm integrating dynamic balanced wavelet coefficients and the Teager energy operator. This approach enhances feature discriminability through multi-step coding reconstruction and attention fusion, and its robustness under strong noise was experimentally validated [6]. Huang et al., tackling the difficulty of effectively eliminating mixed noise in vibration signals of gear transmission systems, proposed a multi-resolution sub-band adaptive filter based on wavelet multi-resolution analysis. By employing a power-of-two variable-order filter structure and a variable step-size optimization algorithm, the noise cancelation capability was improved. Experiments showed its superiority over traditional adaptive filtering methods in enhancing the signal-to-noise ratio (SNR) of various fault features in gears and bearings [7]. Simultaneously, within frameworks based on Empirical Mode Decomposition (EMD/CEEMDAN) and intelligent classifiers, wavelet thresholding is often used as a front-end denoising stage to reduce noise before feature extraction and pattern recognition [8,9,10,11]. However, many of these wavelet-based methods still rely on globally fixed or heuristically set thresholds, which can lead to over-smoothing of fault-related details or insufficient suppression of residual noise, especially when the SNR varies significantly across sub-bands. Yuvaraju et al. [12] combined adaptive threshold wavelet denoising with enhanced ICEEMDAN–Hilbert fusion and an adaptive probabilistic neural network for robust machining chatter detection; Ma Ju et al. [13] incorporated wavelet denoising into CNN-based seismic source localization to improve performance under different noise types; Francesco Melluso et al. [14] employed wavelet-supported residual processing to extract torque fault features in hybrid electric powertrains. Improved thresholding strategies are also seen in laser absorption spectroscopy signal processing [15,16]. Jiang Shuyang et al. [17] applied a method combining correlation-guided IMF selection and adaptive wavelet threshold denoising to enhance the SNR in Raman distributed temperature sensing.

Variational Mode Decomposition (VMD) and its extensions have become important tools for analyzing non-stationary vibration signals. By decomposing a signal into a finite set of band-limited Intrinsic Mode Functions (IMFs), VMD methods can more effectively separate fault-related components from broadband noise compared to classical EMD. However, the performance of VMD is highly dependent on two key parameters: the number of modes (K) and the quadratic penalty factor (α). Improper parameter settings can lead to mode mixing, over-decomposition, or loss of fault information. To address this, many studies have introduced meta-heuristic optimization algorithms to adaptively determine VMD parameters, including sailfish optimization based on the Gini index criterion [18], improved seagull optimization [19], particle swarm optimization [20], and other intelligent optimizers [21,22,23,24,25]. These studies show that parameter-optimized VMD can significantly improve fault feature extraction and noise suppression. However, most existing frameworks use relatively simple objective functions for optimization, which may still suffer from premature convergence or limited global search capability in complex multi-component vibration scenarios. For magnetotelluric data, Wang Zhen et al. combined VMD with mathematical morphological filtering and wavelet thresholding to suppress residual high-frequency noise while better preserving low-frequency components [26].

Successive Variational Mode Decomposition (SVMD), proposed as an improved version of VMD, enhances decomposition adaptability and computational efficiency by extracting IMFs successively. SVMD has been successfully applied to tasks such as underwater acoustic denoising [27], offshore platform modal identification [28], time series prediction [29,30,31], and ship-radiated noise processing [32], often combined with additional criteria like permutation entropy, spectral distance, or correlation coefficients for mode selection and reconstruction [33,34,35]. However, existing SVMD-based denoising frameworks still have two major limitations: (i) SVMD parameters are typically set manually or optimized using general meta-heuristic algorithms, which may not fully exploit the parameter space structure; (ii) when wavelet thresholding is used, fixed or empirically tuned thresholds are often relied upon. These thresholds do not explicitly account for variations in sub-band SNR, making it difficult to balance noise suppression and feature preservation in strongly non-stationary vibration signals.

Meanwhile, recent research in intelligent diagnostics emphasizes the importance of interpretable and noise-robust preprocessing pipelines that provide clean and physically meaningful features for downstream models. Methods based on ICEEMDAN, CEEMDAN, or multi-stage decomposition, combined with optimized thresholds and selection metrics based on energy or kurtosis, demonstrate that well-designed decomposition-denoising chains can significantly improve fault classification accuracy and generalization capability under strong noise [36,37,38]. However, relatively few studies have systematically integrated parameter-optimized SVMD with a structurally adaptive wavelet thresholding strategy specifically designed for non-stationary, multi-component vibration signals of rotating machinery [39,40]. Although SVMD can decompose noisy vibration signals into several band-limited IMFs, the retained informative modes may still contain residual broadband noise, and direct reconstruction may be insufficient to enhance weak impulse-type fault features. Wavelet-based denoising offers a complementary multi-resolution representation well-suited for transient, non-stationary vibration features. Therefore, this paper introduces wavelet thresholding as a second-stage denoising step applied to selected effective IMFs to further suppress residual noise while preserving local fault-related details.

It is noteworthy that in the field of structural health monitoring, several advanced methods for processing non-stationary noisy signals have been developed, offering valuable insights. For instance, Parolai [41], targeting seismogram denoising, proposed a custom thresholding technique based on the S-transform. This method effectively leveraged the S-transform’s advantages of balancing frequency dependency and signal non-stationarity, achieving maximum SNR improvement with minimal information loss, providing a strong reference for handling non-stationary signals. Ditommaso et al. [42] further utilized the adjustable time-frequency resolution of the S-transform to develop a bandwidth-variable filter, successfully extracting time-varying features of specific vibration modes from the non-stationary response of soil and building structures, demonstrating the potential of time-frequency filtering for extracting transient signal components. In a comparative study around the same time, Ditommaso’s team [43] validated the superiority of the S-transform over classical methods and other time-frequency methods (e.g., EMD) for monitoring the dynamic response of masonry towers, avoiding issues like mode mixing. Recently, Zhang et al. [44] addressing dense spatial array data, developed a statistical denoising method based on the curvelet transform. This method effectively separates signal from noise through nonlinear thresholding, improving the SNR while maintaining good waveform consistency and computational efficiency, offering a new approach for processing high-dimensional spatio-temporal data. These studies collectively indicate that strategies combining advanced transform-domain analysis with adaptive thresholding have significant advantages in tackling the challenges of complex noise and non-stationarity.

Wavelet threshold denoising is a commonly used nonlinear denoising technique particularly effective for signals contaminated by random noise. The method decomposes the signal into coefficients across different frequency bands via wavelet transform and then applies thresholding to the high-frequency detail coefficients to suppress noise. The choice of threshold is crucial: a threshold that is too large over-smooths the signal, while one that is too small leaves residual noise. Traditional methods often use a fixed global threshold, which becomes limiting when noise distribution in the signal is non-uniform. Therefore, this paper proposes an adaptive threshold modulation strategy based on sub-band SNRs, dynamically adjusting the threshold according to the SNR of each sub-band to achieve more precise noise suppression and feature retention under strong noise or poor signal quality conditions.

The Cordyceps Fungus Optimization (CFO) algorithm is a novel meta-heuristic optimization algorithm inspired by the parasitic and foraging behavior of cordyceps fungi. It possesses strong global search capability and a good balance between global exploration and local exploitation, making it suitable for high-dimensional complex optimization problems. To further enhance its performance, this study introduces an Improved Cordyceps Fungus Optimization (ICFO) algorithm incorporating Chebyshev chaotic initialization, a longitudinal-transverse crossover fusion mutation operator, and a mind innovation strategy. These enhancements strengthen the algorithm’s global search ability, help avoid local optima, and accelerate convergence. The ICFO is used to optimize the key parameters of SVMD, thereby enhancing the adaptive decomposition capability for complex non-stationary vibration signals.

Based on the above analysis, this paper proposes a joint denoising framework integrating Improved Cordyceps Fungus Optimization (ICFO), SVMD, and an improved wavelet thresholding scheme. First, the ICFO algorithm with Chebyshev chaotic initialization, longitudinal-transverse crossover fusion mutation, and mind innovation strategy is designed to adaptively optimize the penalty factor and mode number of SVMD. Then, the optimized SVMD is used to decompose the noisy vibration signal into a series of IMFs. Next, based on the Pearson correlation coefficient with the original signal, the IMFs are classified into effective components and noise-dominant components. Subsequently, an adaptive wavelet thresholding function modulated by the sub-band SNR is employed to process the effective IMFs. Finally, the denoised signal is reconstructed from the processed components. Simulation studies and experimental results on bearing and gearbox vibration signals demonstrate that the proposed ICFO–SVMD–Improved Wavelet Threshold Denoising (ICFO-SVMD-IWTD) method achieves excellent noise suppression and feature preservation, especially under low SNRs and strong non-stationary conditions.

The highlights of this work are as follows: We propose an ICFO–SVMD–improved wavelet threshold joint denoising framework that achieves adaptive optimization of both SVMD parameters and sub-band thresholds for nonlinear, non-stationary vibration signals; simulations and bearing/gearbox experiments consistently show that this method better preserves transient fault features while improving the SNR/reducing the root mean square error and exhibits stronger robustness (lower relative variance reduction and higher signal enhancement ratio).

The remainder of this paper is organized as follows: Section 2 introduces the improved wavelet threshold denoising method and the ICFO-based SVMD parameter optimization. Section 3 reports the simulation and experimental verification. Section 4 concludes the paper.

## 2. Materials and Methods

**Figure 1:**
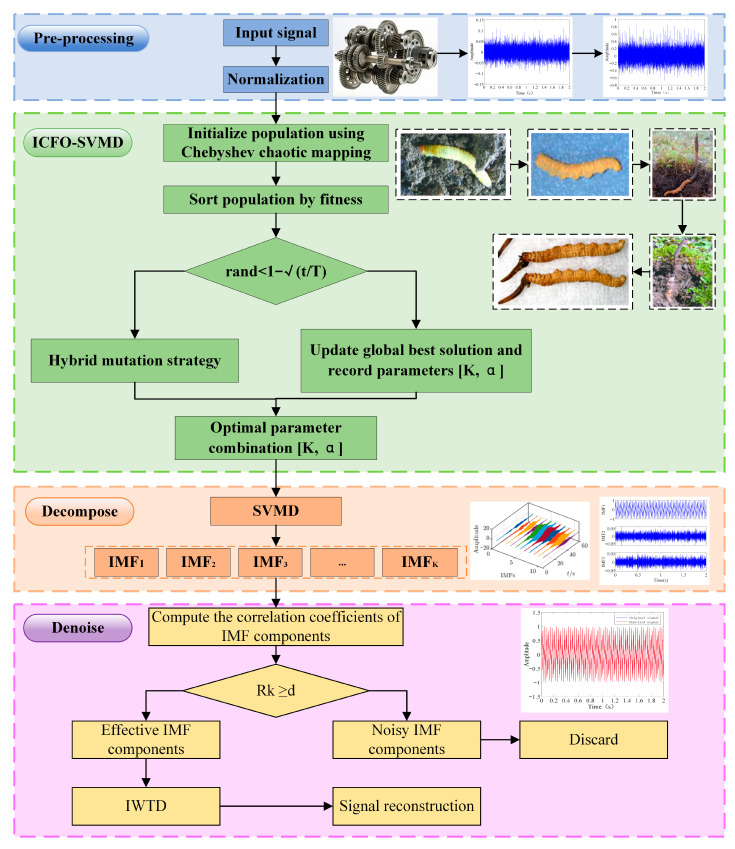
ICFO-SVMD-IWTD framework.

This section presents the methodology of the proposed ICFO–SVMD–WTD denoising framework. Section 2.2 describes the improved wavelet-thresholding strategy, including the sub-band SNR-modulated threshold and the continuous soft–hard transition function. Section 2.3 summarizes SVMD and the improved CFO (ICFO) algorithm used to adaptively optimize the key SVMD parameters (K and α). The complete end-to-end algorithmic pipeline and its explicit correspondence to the flowchart are provided in Section 2.1 (see Figure 1).

### 2.1. ICFO–SVMD–IWTD Algorithm Framework

The overall framework of the proposed ICFO–SVMD–IWTD method is illustrated in Figure 1. First, the input vibration signal is pre-processed to improve subsequent decomposition stability. Then, ICFO is employed to adaptively search for the optimal SVMD parameter pair [K,α]. Specifically, the population is initialized using Chebyshev chaotic mapping and ranked by fitness (minimum envelope entropy). During iterations, a probability-controlled update mechanism is used to switch between the hybrid mutation strategy and the global-best updating/recording process, thereby balancing global exploration and local exploitation and finally yielding the optimal parameter combination [K,α]. Next, SVMD with the optimized parameters decomposes the noisy signal into K intrinsic mode functions (IMFs). After that, the Pearson correlation coefficient between each IMF and the original signal is calculated; IMFs satisfying $R_{k}\geq d$ are regarded as effective components, whereas the remaining noise-dominated IMFs are discarded. Finally, the improved wavelet threshold denoising (IWTD) is applied to the retained effective IMFs and the denoised signal is reconstructed by summing the processed components.

The specific steps are summarized as follows:

**Step 1:** Acquire the original signal.

**Step 2:** Use the minimum envelope entropy as the fitness function for ICFO-based SVMD parameter optimization and adaptively search for the two key SVMD parameters—the number of decomposition modes Kand the penalty factor α—to obtain the optimal parameter pair [K,α].

**Step 3:** Decompose the noisy signal using SVMD with the optimized parameter pair [K, α] and obtain a set of intrinsic mode functions (IMFs).

**Step 4:** Compute the Pearson correlation coefficient between each IMF component and the original signal. According to the magnitude of the Pearson correlation coefficient, the IMFs are divided into effective components and noise components.

(1) $$R_{k}=\frac{\sum_{i=1}^{n}\left[x(t)-\bar{x}\right]\left[u_{k}(t)-\bar{u_{k}}\right]}{\sqrt{\sum_{i-1}^{n}\left[x(t)-\bar{x}\right]\sum_{i=1}^{n}{\left[x(t)-\bar{x}\right]}^{2}}}$$

where x(t) represents the original signal, $u_{k}(t)$ is the k−th IMF component, $\bar{x}$ and $\bar{u_{k}}$ are their respective mean values, and n is the signal length.

**Step 5:** Apply the improved wavelet thresholding function to denoise the effective IMF components.

**Step 6:** Reconstruct the signal from the denoised components to obtain the final denoised signal.

### 2.2. Improved Wavelet Threshold Denoising Method

Wavelet threshold denoising is a widely used nonlinear denoising technique used for signals corrupted by additive noise [45]. In general, it consists of three steps: (i) performing a discrete wavelet transform (DWT) to obtain multi-scale approximation and detail coefficients; (ii) shrinking or discarding the detail coefficients whose magnitudes are mainly contributed by noise using a thresholding rule; and (iii) reconstructing the signal via the inverse DWT. Compared to conventional linear filters, this coefficient-domain shrinkage can achieve an effective trade-off between noise suppression and the preservation of transient structures in non-stationary vibration signals.

In practical engineering applications, the acquired signals are usually contaminated by various types of random noise, and their relationship is generally described by the additive noise model in Equation (2):

(2) y(t) = x(t) + n(t)

where x(t) denotes the true useful signal and n(t) denotes the noise term.

Conventional linear filtering methods in the time- or frequency-domain exhibit limitations when processing non-stationary multi-component signals. They often fail to effectively suppress noise while simultaneously preserving the key local time–frequency features of the signal.

The wavelet transform, which has favorable time–frequency localization characteristics, enables multiscale decomposition of the signal, whereby the low-frequency components represent the overall trend of the signal and the high-frequency components characterize detailed and abrupt features. On this basis, Donoho and Johnstone proposed the concept of wavelet threshold denoising: the noisy signal is first decomposed by a wavelet transform, then a thresholding operation is applied to the high-frequency coefficients in the wavelet-coefficient-domain to suppress those coefficients contributed mainly by noise, and finally the denoised signal is obtained by wavelet reconstruction.

#### 2.2.1. Improved Wavelet Thresholding

The selection of the wavelet threshold directly determines the trade-off between denoising strength and signal fidelity, and is therefore one of the key issues in wavelet thresholding-based denoising. If the threshold is set too large, signal details tend to be excessively smoothed; if it is set too small, significant residual noise will remain. In the classical VisuShrink [46] scheme, a single global “universal threshold” is applied to the wavelet detail coefficients at all scales, typically chosen as

(3) $$\lambda =\sigma \sqrt{2\ln N}$$

where σ is the standard deviation of the noise, which is usually estimated from the median of the high-frequency sub-band coefficients, and N is the length of the signal.

This threshold rule is derived under the assumption of i.i.d. Gaussian noise and has asymptotic near-minimax performance, which makes it robust and easy to implement; however, it is also known to be conservative and may over-smooth fine details in practical vibration signals. In this work, the proposed sub-band SNR-modulated threshold can be viewed as a refinement of the VisuShrink philosophy: it retains the robustness of the universal threshold in noise-dominated sub-bands while reducing unnecessary shrinkage in structure-dominated sub-bands [46,47]. The universal threshold can achieve asymptotically optimal mean square error performance under the assumption of Gaussian white noise, and therefore exhibits good robustness. However, this threshold is evidently conservative with respect to noise suppression and tends to weaken fine details and blur edges in practical signal processing, especially for vibration signals with rich structural components.

To overcome this drawback while maintaining the robustness of the universal threshold and improving structural fidelity, an adaptive attenuation strategy is introduced within the framework of a fixed threshold to modulate the original threshold. The improved threshold is given by

(4) $$\lambda^{*}=\alpha \sigma \sqrt{2\ln N}$$

where α is an adjustment coefficient with value (0,1] used to control the effective magnitude of the threshold under different local structural conditions and thus realize adaptive adjustment of the threshold. $\lambda^{*}$ denotes the modified threshold.

In recent years, the globally fixed threshold adopted in conventional VisuShrink has proved inadequate for accommodating the variations in signal/noise energy distribution across different frequency bands. Consequently, related studies have introduced the concept of sub-band-adaptive thresholds in the wavelet-domain, whereby the noise variance and signal variance are estimated separately within each sub-band and the threshold is dynamically adjusted according to local statistical characteristics. Building on this idea, the present study further expresses the estimated noise and signal energies in each sub-band in terms of a sub-band signal-to-noise ratio (SNR) [47]. Let the total energy of the coefficients in a given sub-band ω be

(5) $$\upsilon =E[\omega^{2}]$$

where ω denotes the noisy wavelet coefficients in the sub-band.

The corresponding signal energy can be estimated as

(6) $${\hat{\sigma }}_{x}=\sqrt{\max(\upsilon -{\hat{\sigma }}^{2},0)}$$

where ${\hat{\sigma }}_{x}$ denotes the estimated standard deviation of the underlying (true) signal component in the sub-band. $\hat{\sigma }$ denotes the estimated noise standard deviation, which is calculated by

(7) $$\hat{\sigma }=\frac{Median(|X|)}{0.6745}$$

where X is the sample set used for noise estimation and Median(|X|) is the median of |X|.

Accordingly, a normalized SNR factor is constructed as

(8) $$\eta =\frac{{\hat{\sigma }}^{2}}{{\hat{\sigma }}^{2}+{\hat{\sigma }}_{x}^{2}}\in [0,1]$$

where η≈1 characterizes the degree to which the sub-band is dominated by noise; a smaller η indicates that the structural component in this sub-band is stronger.

To achieve adaptive adjustment for different structural strengths, the following scaling relation is adopted:

(9) $$\alpha =\eta^{\beta },\;0.5\leq \beta \leq 1$$

where β is the exponent that controls the adaptation strength: the larger this value is, the more “linear” α varies with η; conversely, a smaller β leads to a more “moderate” variation.

Using the above procedure, when a sub-band is dominated by noise (i.e., when η≈1 indicates a noise-dominated sub-band), the threshold remains almost unchanged, thereby preserving the strong noise-suppression capability of the original universal threshold. In contrast, when the sub-band contains pronounced signal structures, the threshold is accordingly reduced, which effectively prevents excessive attenuation of fine details. Compared with conventional fixed-threshold strategies, the proposed method achieves a more favorable trade-off between noise suppression and structural fidelity.

#### 2.2.2. Improved Wavelet Threshold Function

Conventional wavelet thresholding methods can be broadly classified into soft and hard thresholding [48]. Soft thresholding offers good continuity and helps to avoid artifacts, but introduces a systematic bias for coefficients with large amplitudes. Hard thresholding, on the other hand, is unbiased for large coefficients, but exhibits a discontinuity at the threshold, which readily leads to ringing and artifacts. Therefore, designing an improved threshold function that preserves the advantages of both approaches while ensuring that good continuity, low bias, and tunability is an important means to enhance the quality of wavelet-based denoising.

Motivated by this, an improved threshold function with a “continuous soft–hard transition” characteristic is proposed in this study. The threshold function is defined as follows:

(10) $$f(\omega ;\lambda ,b)=\left\{\begin{array}{ll} 0, & |\omega |<\lambda \\ \omega -sign(\omega )\frac{\lambda }{\exp(b(|\omega |-\lambda ))}, & |\omega |\geq \lambda \end{array}\right.$$

where λ is the threshold parameter, b is the adjustment factor controlling the strength of coefficient shrinkage, and ω denotes the original wavelet coefficient.

**Figure 2:**
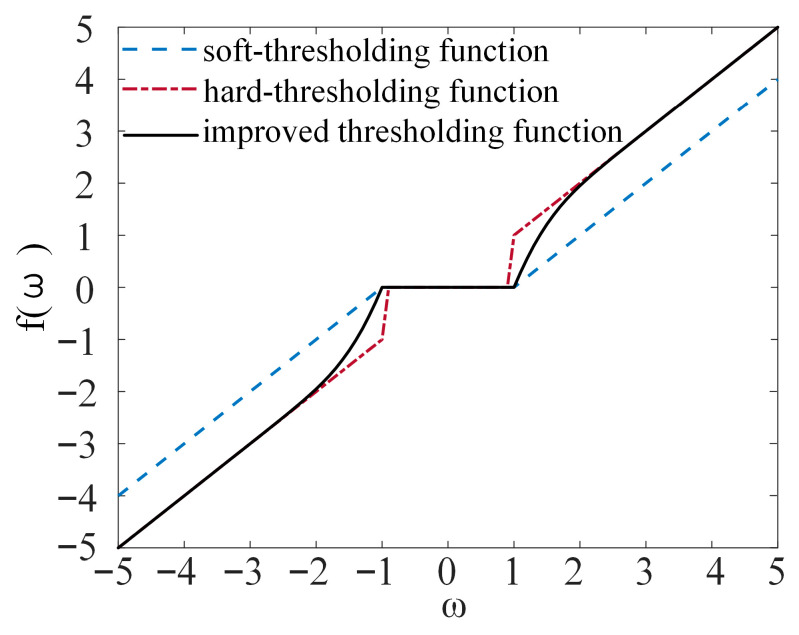
Threshold function curve.

The curves of the conventional soft and hard threshold functions, together with the proposed improved wavelet threshold function, are shown in Figure 2.

### 2.3. Optimization of SVMD Parameters Based on Improved CFO

#### 2.3.1. Principles of Successive Variational Mode Decomposition

Successive variational mode decomposition (SVMD) is an adaptive signal processing method proposed by M. Nazari et al. in 2020 [49]. Built upon the framework of variational mode decomposition (VMD), this method further refines the constraint conditions and solution procedure. SVMD does not require prior specification of key parameters, such as the number of modes or their center frequencies, and can automatically decompose a complex multi-component signal into a series of mono-component intrinsic mode functions, each representing a specific frequency component of the signal.

By successively extracting modes from the time series and gradually reconstructing the original signal, SVMD not only improves the adaptivity of the decomposition, but also significantly reduces computational complexity, making it suitable for the analysis and processing of various non-stationary signals. The basic principle of the SVMD algorithm is as follows:

(1) For a time series f(t), it is assumed that it can be decomposed as

(11) $$f(t)=u_{L}(t)+f_{r}(t)$$

where $u_{L}(t)$ denotes the L−th mode and $f_{r}(t)$ is the residual signal.

(2) To ensure that the assumption in (1) can be satisfied, a minimization constraint for the *L* modes is constructed, as given in Equation (12).

(12) $$J_{1}=\left\|\partial_{t}\left[\left(\delta (t)+\frac{j}{\pi t}\right)*u_{L}(t)\right]e^{\;\text{-}\;j\omega_{L}t}\right\|$$

where $\partial_{t}$ denotes the Dirac delta function, δ(t) is the impulse function, j is the imaginary unit, ∗ is the convolution operator, $\omega_{L}$ is the center frequency of the L−th mode, and $e^{-j\omega_{L}t}$ is the rotation factor.

(3) To guarantee the stable realization of the constraint in Equation (12), an appropriate filter is selected, whose frequency response is required to satisfy

(13) $${\hat{\beta }}_{L}(\omega )=\frac{1}{\alpha {\left(\omega -\omega_{L}\right)}^{2}}$$

where ω is the center frequency of the impulse response and α is a balancing parameter. Accordingly, a second constraint can be formulated as

(14) $$J_{2}={\left\|\beta_{L}(t)*f_{r}(t)\right\|}_{2}^{2}$$

where $\beta_{L}(t)$ is the impulse response of the filter described by Equation (13).

(4) With only the two constraints in Equations (12) and (14), the L−th-order mode and the L−1−th-order mode cannot be effectively distinguished. Therefore, following the idea used to construct constraint $J_{2}$, a filter is selected whose frequency response is required to satisfy

(15) $${\hat{\beta }}_{i}(\omega )=\frac{1}{\alpha {\left(\omega -\omega_{L}\right)}^{2}},i=1,2,\ldots ,L-1$$

Thus, a third constraint can be established as

(16) $$J_{3}=\sum_{i}^{L-1}{\left\|\beta_{i}(t)*u_{L}(t)\right\|}_{2}^{2}$$

where $\beta_{i}(t)$ denotes the frequency-domain impulse response.

(5) To ensure that the signal can be perfectly reconstructed during the decomposition process, the following constraint should also be imposed:

(17) $$f(t)=u_{L}(t)+f_{u}(t)+\sum_{i=1}^{L-1}u_{i}(t)$$

On the basis of the above analysis, the problem of extracting modal components can thus be formulated as a constrained minimization problem:

(18) $$\left\{\begin{array}{l} \underset{u_{L},\omega_{L},f_{r}}{\min}\left(\alpha J_{1}+J_{2}+J_{3}\right)\\ s.t.\;u_{L}(t)+f_{r}(t)=f(t) \end{array}\right.$$

where α is a parameter used to balance $J_{1},J_{2}\;\mathrm{and}\;J_{3}$.

#### 2.3.2. Principle of the Cordyceps Fungus Optimization Algorithm

The cordyceps fungus optimization algorithm is a novel metaheuristic algorithm inspired by the life process of cordyceps. It simulates the unique foraging and parasitic behavior of cordyceps, whose core advantages lie in its strong global search capability and its effective strategies for avoiding local optima. By means of efficient exploration operators, the algorithm achieves a good balance between global exploration and local exploitation. In addition, the introduced “re-parasitism” and “optimal parasitism” mechanisms further emulate the parasitic characteristics of cordyceps, significantly enhancing the ability to escape local optima and thereby ensuring the robustness of the search process [50].

(1) Exploration operator

In the exploration phase, the cordyceps population is first sorted in descending order according to fitness. Subsequently, each individual randomly selects one of two exploration mechanisms to update its position, and the two mechanisms are chosen with equal probability.

1. Wave advance operator

When an individual selects the wave advance operator, its search process is designed to mimic a wave-like advancing dynamic behavior. The mathematical model of this behavior can be described as

(19) $$X_{CF,i}^{SO}=\left\{\begin{array}{ll} X_{CF,i}\;\text{-}\;\mathrm{rand}(1,\mathrm{Dim})*(X_{best}\;\text{-}\;X_{CF,i})+\mathrm{alpha}*(X_{best}\;\text{-}\;X_{CF,i}), & i=1\\ X_{CF,i}\;\text{-}\;\mathrm{rand}(1,\mathrm{Dim})*(X_{CF,i\text{-}1}\;\text{-}\;X_{CF,i})+\mathrm{alpha}*(X_{best}\;\text{-}\;X_{CF,i}), & i=2,3,\ldots ,N \end{array}\right.$$

where $X_{CF,i}^{SO}$ denotes the position of the i−th cordyceps individual in the search phase, Dim is the number of decision variables, $X_{best}$ is the position of the cordyceps individual with the minimum fitness value, and $X_{CF,i-1}$ and $X_{CF,i}$ represent the positions of the i−1 and i−th cordyceps individuals, respectively. Since the cordyceps individuals are sorted in descending order of fitness, the i−1−th cordyceps individual performs better than the i−th one. alpha is defined as

(20) alpla=2.5∗rand(1,Dim)∗|cos(π∗rand(1,Dim))|

2. Spiral rising operator

In the spiral rising operator stage, the search mechanism adopted by the cordyceps is described by the following mathematical Equation (21):

(21) $$X_{CF,i}^{SO}=\left\{\begin{array}{ll} X_{best,i}\;\text{-}\;rand(1,Dim)*(X_{best}\;\text{-}\;X_{CF,i})+beta*(X_{best}\;\text{-}\;X_{CF,i}), & i=1\\ X_{best,i}\;\text{-}\;rand(1,Dim)*(X_{CF,i\text{-}1}\;\text{-}\;X_{CF,I})+beta*(X_{best}\;\text{-}\;X_{CF,i}), & i=2,3,\ldots ,N \end{array}\right.$$

where beta denotes the search step size of the cordyceps at this stage, which is calculated as follows:

(22) $$beta=2*\cos(\pi *r_{1})*\left|{\left(\frac{it}{Max\text{\_}iteration}\right)}^{r_{1},r_{2}}\right|$$

where $r_{1}$ is a random number uniformly distributed in [0, 1], $r_{2}$ is a random integer taking the value 1 or 2, $r_{2}$ is randomly selected for the whole population, it is the current iteration number, and Max_iteration is the maximum number of iterations. ${\left(\frac{it}{Max\text{\_}iteration}\right)}^{r_{1},r_{2}}$ denotes the search radius of the cordyceps individual, which is used to perform local search in its neighborhood to locate the optimal solution. When $r_{2}=2$ takes a small value, the cordyceps conducts a detailed search within a small region. Moreover, as the number of iterations gradually approaches the maximum, this parameter tends to 1. Such a gradual adjustment ensures that, if the search becomes trapped in a local optimum, increasing $\frac{it}{Max\text{\_}iteration}$ can enhance the algorithm’s ability to escape that local optimum in the later stages of the iterations.

(2) Larval parasitic process

The CFO algorithm guides population updating by mimicking the strategic choice of cordyceps between “optimal parasitism” and “re-parasitism”. The mathematical modeling of this selective parasitic behavior effectively enhances the algorithm’s ability to converge towards solutions with a higher success rate.

1. Re-parasitic behavior

In this stage, cordyceps individuals continue to exploit the resources of the same host larva. To improve parasitic reliability, individuals with better fitness will perform “re-parasitism” on that larva. This process is expressed as

(23) $$X_{CF,i}^{pl}=X_{CF,i}^{SO}+3*r_{3}*\left\{\mathrm{rand}*X_{best}-\mathrm{rand}*X_{CF,i}^{SO}\right\},\;i=1,2,\ldots ,N$$

where $X_{CF,i}^{pi}$ denotes the position of the i-th cordyceps individual in the parasitic phase, $X_{CF,i}^{SO}$ is its position in the exploration phase, and $r_{3}$ is a random scalar drawn from a standard normal distribution. Since $r_{3}$ is most likely to be close to zero, the resulting displacement is small in most cases; however, in a few cases, $r_{3}$ may take a relatively large value, leading to a pronounced displacement that drives the cordyceps into new search regions and helps the algorithm escape from local optima.

2. Optimal parasitic behavior

In contrast to the “re-parasitic behavior” that targets the same host, “optimal parasitic behavior” mainly occurs when a cordyceps individual encounters a larva that has already been parasitized by other individuals. At this moment, the core of its strategy shifts to actively searching for globally superior growth conditions, and its behavioral rule is defined as follows:

(24) $$X_{CF,i}^{pl}=X_{\mathrm{best}}+lamda*\left\{\mathrm{rand}*X_{best}-\mathrm{rand}*X_{CF,i}^{SO}\right\},\;i=1,2,\ldots ,N$$

The parameter lamda is calculated as

(25) $$lamda=r_{4}*\left\{{\left(\frac{\mathrm{it}}{{\mathrm{Max}}_{\text{-}}\mathrm{iteration}}\right)}^{2}-2*\frac{\mathrm{it}}{{\mathrm{Max}}_{\text{-}}\mathrm{iteration}}+1\right\}$$

where $r_{4}$ is a random number uniformly distributed in [0,1]. The parameter lamda allows for the cordyceps individuals to take relatively large search steps in the early iterations. As the number of iterations increases, lamda gradually approaches zero, thereby achieving a smooth transition from large-scale exploration to small-scale exploitation.

#### 2.3.3. Improvements to the Cordyceps Fungus Optimization Algorithm

To further enhance the global optimization ability and convergence accuracy of the original CFO, three improvement mechanisms are introduced into the original “wave advance–spiral rising–re-parasitism–optimal parasitism” framework, namely a Chebyshev chaotic mapping–based initialization strategy, a longitudinal–transverse crossover fusion mutation strategy, and a Thinking Innovation Strategy (TIS). These improvements respectively strengthen the diversity of the initial population, the ability to escape from local optima in the middle search stage, and the convergence stability in the later stage, thus achieving an adaptive balance between global exploration and local exploitation. The improved principles are described below.

(1) Chebyshev Chaotic Initialization Strategy

In the original CFO algorithm, uniform random initialization is adopted, which often results in an uneven distribution of individuals in the search space and insufficient diversity in the early search stage. To enhance the coverage of the initial population, Chebyshev chaotic mapping is employed for initialization in this study. Owing to its ergodicity and pseudo-random characteristics, this mapping enables the search individuals to be more uniformly distributed over the entire search space, thereby improving the global exploration capability of the algorithm.

Let the dimension of the search space be D and the population size be N. The chaotic sequence of the i−th individual is generated by the Chebyshev mapping as follows:

(26) $$x_{i,j}^{\left(k+1\right)}=\cos(k\cdot \arccos(x_{i,j}^{\left(k\right)})),\;j=1,2,\ldots ,D$$

where $x_{i,j}^{\left(k+1\right)}\in [-1,1]$ denotes the k-th chaotic iteration value. By normalizing it to the search interva $\left[l_{j},u_{j}\right]$, the initial position of each individual can be obtained as

(27) $$X_{i,j}=l_{j}+(u_{j}-l_{j})\times \frac{x_{i,j}^{\left(k\right)}+1}{2}$$

This strategy effectively enhances the diversity and coverage of the initial solutions, prevents the algorithm from being trapped in local optima at an early stage, and provides a high-quality initial distribution for the subsequent search process.

(2) Longitudinal–transverse Crossover Fusion Mutation Strategy

To further improve the global escape capability and search accuracy of CFO in the middle search stage, a longitudinal–transverse crossover fusion mutation operator is introduced between the exploration and parasitic phases. This strategy combines three mechanisms—global learning, individual perturbation, and Lévy flight—and achieves a dynamic balance through adaptively controlled parameters. The position update formula for the mutation operator is given by

(28) $$X_{i}^{\prime }=r_{1}S_{1}+r_{2}S_{2}+r_{3}S_{3}$$

(29) $$\left\{\begin{array}{l} S_{1}=X_{best}+\alpha (u-l)\cdot N(0,1)\\ S_{2}=X_{i}+(X_{r}-X_{i})\cdot rand()\\ S_{3}=X_{i}+L\cdot (u-l)\cdot e^{-\frac{t}{0.1T}} \end{array}\right.$$

where $r_{1,}r_{2},r_{3}\in (0,1)$ is the weight coefficient, L is the Lévy-distributed step size, and t and T denote the current and maximum iteration numbers.

This strategy enables wide-range global exploration in the early search stage and gradually strengthens local exploitation in the later stage, effectively preventing premature convergence of the algorithm.

(3) Thinking Innovation Strategy

To enhance the local exploitation ability and convergence stability in the later stage of the algorithm, a Thinking Innovation Strategy (TIS) is introduced in this study [51]. By incorporating cognitive perturbation and dynamically adjusted coefficients, this strategy guides individuals to perform “rethinking”-type research in the vicinity of the global optimum so as to achieve superior local exploitation. Its mathematical formulation is given as follows:

(30) $$X_{i}^{new}=\tan(\pi IM-0.5\pi )+\frac{X_{i}}{DOK}+IE$$

where IM=π⋅IE⋅rand(), IE represents the position of the current global best individual, and DOK is a dynamic adjustment factor, rand()∈(0,1).

(31) $$DOK=C+{\left(FES/\max FES\right)}^{C}+FES^{10}$$

By introducing a “thinking leap” in the final stage of the algorithm, this strategy realizes adaptive perturbation around the global optimum, generating new potential solutions in its vicinity and thereby enhancing the convergence accuracy and stability of the CFO algorithm.

#### 2.3.4. Performance Verification of the Improved CFO Algorithm

To verify the effectiveness of the proposed improved CFO algorithm, systematic simulation experiments were carried out in the MATLAB R2023b environment. The population size was set to N=30 and the maximum number of iterations to T=100. A total of 12 standard benchmark functions were selected as performance indicators, including four unimodal functions (F1–F4), five multimodal functions (F5–F9), and three fixed-dimension composite functions (F10–F12), so as to comprehensively evaluate the algorithm on different types of optimization problems. The mathematical expressions, search ranges, and theoretical optima of all test functions are summarized in Table 1.

The experimental results demonstrate that the improved CFO algorithm exhibits superior performance on most test functions. For unimodal functions such as F1 and F2, the algorithm converges stably to the vicinity of the theoretical optimum, and the mean error is significantly lower than that of the compared algorithms, indicating excellent convergence accuracy. For multimodal functions, the improved CFO shows a stronger ability for global exploration and escaping from local optima. In particular, on complex multimodal problems such as F6, its optimization results are markedly better than those of the original CFO and classical algorithms like GWO, highlighting its enhanced robustness.

**Figure 3:**
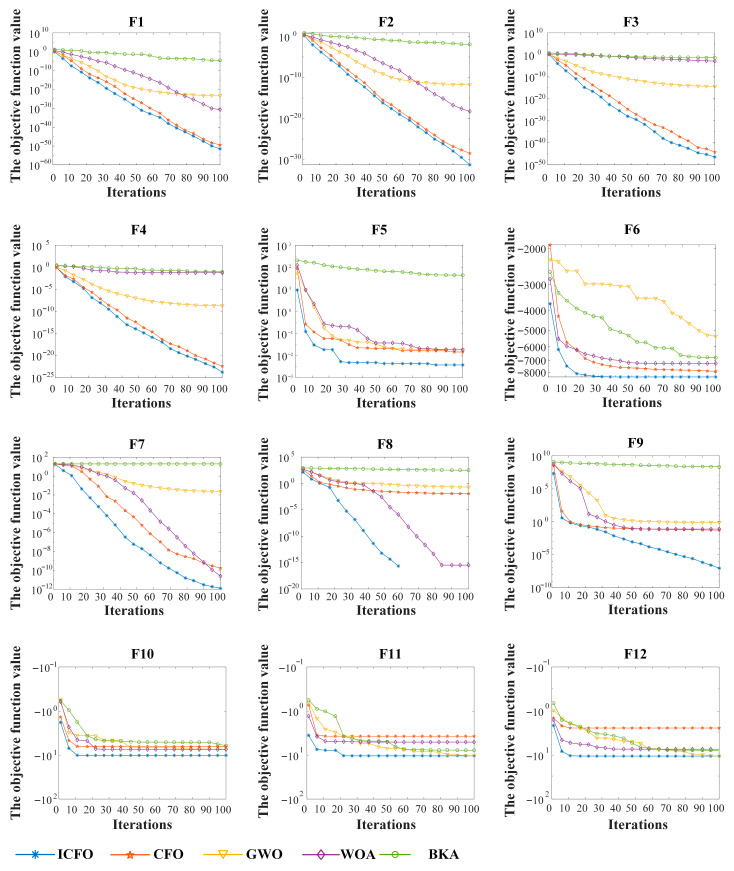
Optimization algorithm iteration curve graph.

Overall, based on the numerical results in Table 2 and the convergence curves shown in Figure 3, the improved CFO algorithm exhibits clear advantages in solution accuracy, convergence speed, and stability. It effectively alleviates the limitations of traditional optimization algorithms, such as premature convergence and low search efficiency, when dealing with complex multimodal and high-dimensional problems. These improvements are mainly attributed to the three introduced strategies, which increase population diversity in the early stage of the search, enhance global exploration and local escape capabilities in the middle stage, and realize smoother and faster convergence in the later stage.

#### 2.3.5. Procedure for Optimizing SVMD Parameters with the Improved CFO Algorithm

SVMD is an advanced signal decomposition method whose performance largely depends on the selection of two key parameters: the penalty factor, α, and the number of modes, K. If these two parameters are improperly set, problems such as mode mixing, over-decomposition, or under-decomposition may occur, thereby degrading the accuracy of the decomposition results. Traditional parameter selection methods typically rely on empirical trial-and-error, which is inefficient and cannot guarantee that the optimal solution will be obtained. Therefore, in this study, the improved CFO algorithm is employed to optimize the values of K and α in SVMD so as to rapidly and accurately determine the optimal parameter combination.

**Figure 4:**
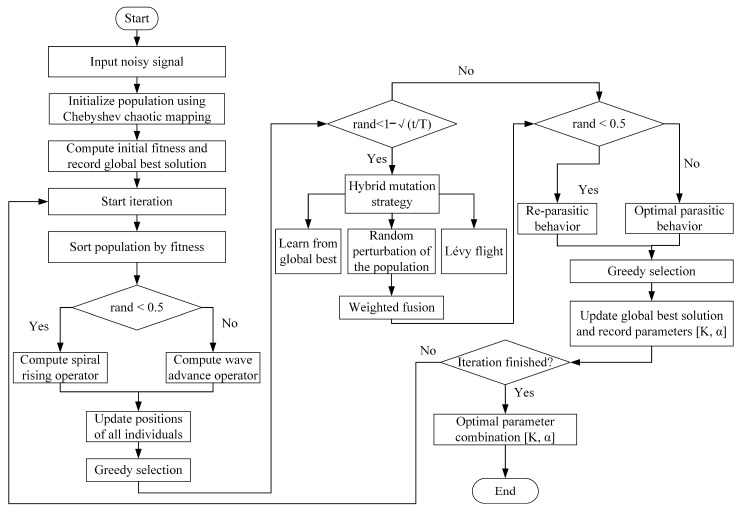
The process of how ICFO optimizes SVMD.

By leveraging the advantages of the improved CFO algorithm, an ICFO-SVMD signal decomposition method based on CFO is proposed. Through the incorporation of Chebyshev chaotic mapping, the longitudinal–transverse crossover fusion mutation strategy, and the Thinking Innovation Strategy, the global search capability of the algorithm is strengthened and the trapping in local optima is avoided, enabling efficient exploration and precise convergence in the parameter space. In this way, the optimal K and α for SVMD can be obtained, ensuring that the signal is effectively decomposed. The overall procedure of the ICFO -SVMD signal decomposition is illustrated in Figure 4.

**Step 1: Initialize algorithm parameters and population**

Set the population size and maximum number of iterations for the improved Cordyceps Fungus Optimization (ICFO) algorithm and determine the search range for the key SVMD parameters (the number of modes K and penalty factor α). Use the Chebyshev chaotic mapping strategy to initialize the population positions, enhancing the diversity of the initial solutions.

**Step 2: Iterative optimization of SVMD parameters**

Use the minimum envelope entropy as the fitness function to evaluate the performance of the SVMD signal decomposition for the current parameter combination. The ICFO algorithm dynamically adjusts the position of each individual in the population (i.e., a set of candidate parameters) based on the fitness values, utilizing exploration operators such as wavefront advancement and spiral ascent, along with an update mechanism incorporating cross-over mutation and innovative strategies to continuously search for better parameter combinations.

**Step 3: Check termination condition and output optimal parameters**

Repeat the iterative process in Step 2 until the maximum number of iterations is reached or the fitness value converges to stability. Finally, output the optimal parameter combination K and α obtained using global optimization by ICFO, which will be used for precise decomposition of the subsequent SVMD signals.

## 3. Experimental Verification

### 3.1. Simulation-Based Verification

#### 3.1.1. Rotating Machinery Vibration Signal Simulation Verification

To verify the effectiveness of the proposed method for the analysis of vibration signals in rotating machinery, simulation experiments were carried out on a synthetic vibration signal in the MATLAB 2023b environment. Vibration signals of rotating machinery usually contain multiple complex noise components arising from background noise, component friction, and external disturbances. To ensure that the simulation conditions are close to practical engineering applications, a composite simulation signal containing several typical fault features was constructed for testing as follows:

(32) $$\left\{\begin{array}{l} x_{1}(t)=0.25\cos(42.5\pi t)\\ x_{2}(t)=0.3(1+1.5\sin(20\pi t))\sin(100\pi t)\\ x_{3}(t)=0.15e^{-15t}\sin(200\pi t)\\ X(t)=x_{1}(t)+x_{2}(t)+x_{3}(t)+noise \end{array}\right.$$

where t denotes the time variable. In this simulated signal, component $x_{1}(t)$ represents the simple harmonic vibration of the rotor, component $x_{2}(t)$ is a typical amplitude-modulated signal used to emulate the periodically impact-modulated phenomenon caused by rolling bearing faults and similar defects, and component $x_{3}(t)$ is a decaying oscillation used to simulate the high-frequency natural vibration excited by instantaneous impacts of mechanical components. Finally, a noise term is added to mimic noise interference under actual operating conditions.

**Figure 5:**
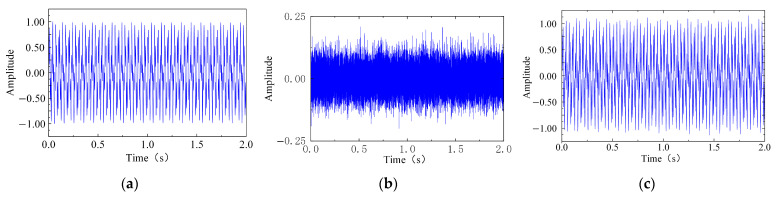
Nonlinear, non-stationary simulated signal with an SNR of 20 dB, (**a**) clean signal, (**b**) 20 dB Gaussian white noise, and (**c**) simulated signal with noise.

The above signal combination can effectively reproduce the coexistence of multiple fault features and complex noise in practical vibration signals. To validate the applicability and superiority of the proposed ICFO–SVMD–improved wavelet threshold joint denoising method for nonlinear, non-stationary vibration signals, the constructed simulation signal is taken as the study object, and comparative experiments are performed under different noise levels with SNRs = 1 dB, 5 dB, 10 dB, 15 dB, and 20 dB. Taking the 20 dB noise condition as an example, the sampling frequency was set to 12,800 Hz, the number of sampling points was 25,600, and the signal duration was 2 s. The time-domain waveforms of the original signal and the signal contaminated with additive Gaussian white noise are shown in Figure 5. It can be seen that the addition of noise markedly masks the periodic impacts and decaying oscillatory components, leading to a significant degradation in signal clarity.

**Figure 6:**
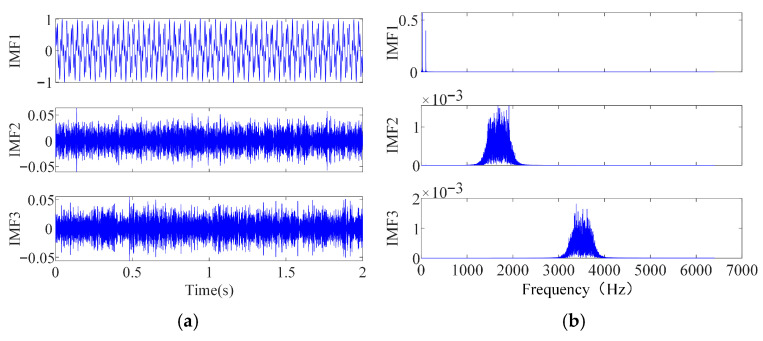
IMF components’ time-domain and frequency-domain plots. (**a**) Time-domain plot of IMF. (**b**) Frequency-domain plot of IMF.

The improved cordyceps fungus optimization algorithm is then employed to adaptively optimize the key SVMD parameters, namely the penalty factor α and the number of modes K, where the search range of K is set to [2,10] and that of α to [1000,3000]. After ICFO, the optimal parameter pair [9,2736] is obtained. Substituting this optimal pair into SVMD, the noisy simulation signal is decomposed into several intrinsic mode functions (IMFs). The time-domain waveforms and spectra of each IMF component are shown in Figure 6.

The Pearson correlation coefficient between each IMF component and the original noise-free signal is then calculated, as summarized in Table 3. Using a correlation coefficient threshold as the decision criterion, IMFs with a high correlation are regarded as effective components, whereas the remaining modes are treated as noise-dominated components and discarded. The improved wavelet thresholding method is applied to the retained effective IMFs for denoising, and the final denoised signal is obtained by reconstructing these processed components.

**Figure 7:**
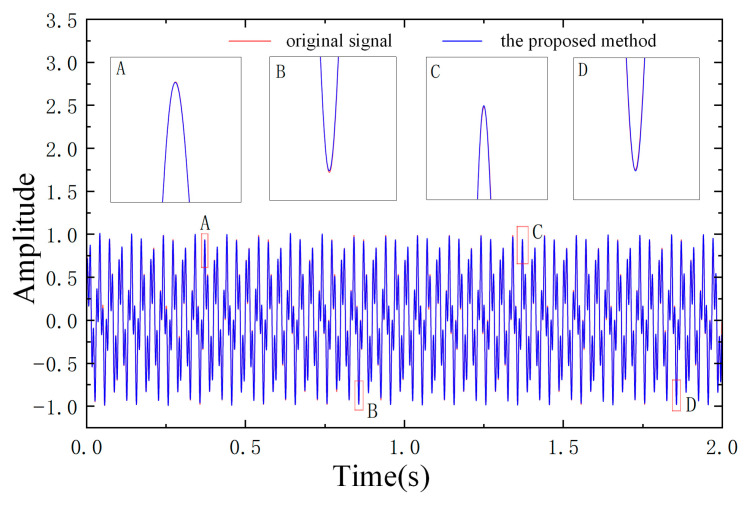
Comparison chart of noise-reduced signal and original signal.

The time-domain waveforms of the original signal and the signal processed by the ICFO–SVMD–improved wavelet threshold denoising algorithm are compared in Figure 7. As can be observed from Figure 7, after denoising, the periodic characteristics and high-frequency oscillatory components of the signal become much clearer, the noise is effectively suppressed, and the key information is well preserved.

To verify the accuracy of the results shown in Figure 7, the root mean square error (RMSE) and residual variance ratio (RVR) between the original signal and the denoised signal were calculated. The calculated RMSE values show a significant reduction in error compared to other methods, further validating the effectiveness of the ICFO-SVMD-IWTD joint denoising method. The detailed results can be found in Table 4.

**Figure 8:**
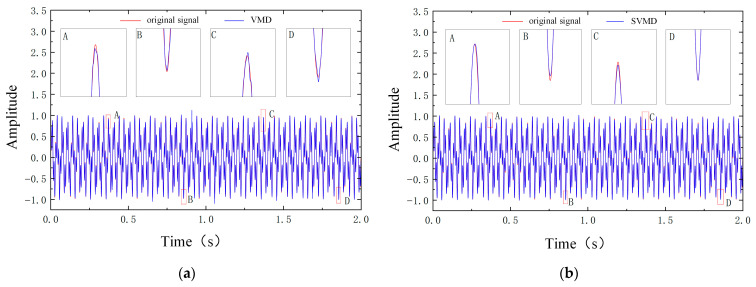

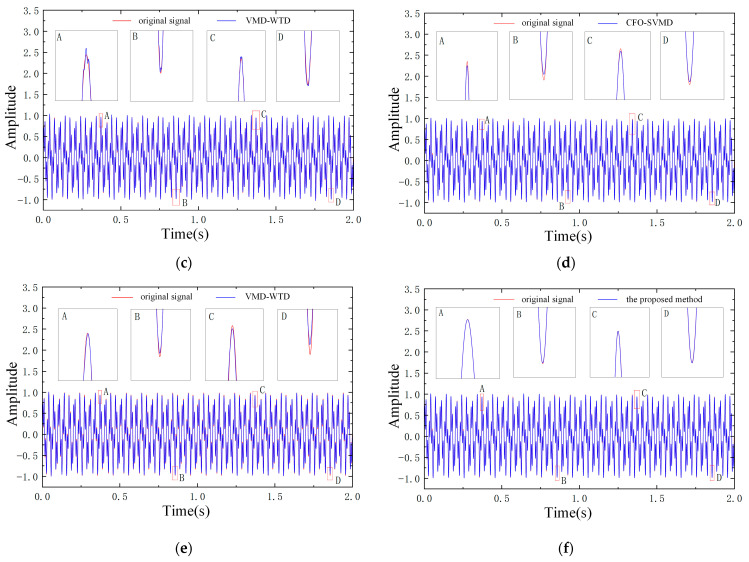
Denoising effect plots of different denoising methods. (**a**) Denoising results of the VMD algorithm. (**b**) Denoising results of the SVMD algorithm. (**c**) Denoising results of the VMD-WTD algorithm. (**d**) Denoising results of the CFO-SVMD algorithm. (**e**) Denoising results of the WTD algorithm. (**f**) Denoising results of the proposed algorithm.

To further quantitatively evaluate the denoising performance of the proposed method, it is compared with several classical algorithms, including the conventional wavelet thresholding method, VMD, SVMD, CFO-SVMD, and the VMD–wavelet method without parameter optimization, as shown in Figure 8. By examining the time-domain waveforms of the noisy simulation signal processed by different denoising algorithms in Figure 8, the performance differences among the methods can be visually assessed.

Compared to the other five methods, the signal processed by the proposed ICFO–SVMD–improved wavelet threshold method exhibits an overall waveform that is closest to the original clean signal, demonstrating superior noise suppression and feature preservation. Inspection of the enlarged regions A, B, C, and D reveals the following: the signal processed by the traditional wavelet thresholding method is globally smooth, but suffers from a pronounced attenuation of impact amplitudes; the signals reconstructed from VMD or SVMD still contain a considerable amount of high-frequency spike-like noise; and although the combined methods such as VMD–WTD and CFO–SVMD improve the denoising performance to some extent, waveform distortion or blurred details remain around the impact peaks. In contrast, the signal obtained by the proposed method preserves the amplitude of the impact components, renders the transient high-frequency oscillations clearly distinguishable, and maintains a stable baseline with almost no residual noise fluctuations.

#### 3.1.2. TDLAS Second Harmonic Signal Simulation Verification

To further validate the generality of the proposed method in different types of signals, this study constructs a TDLAS (Tunable Diode Laser Absorption Spectroscopy) second harmonic simulation signal for testing. TDLAS technology is commonly used for gas concentration detection, and its second harmonic signal is susceptible to Gaussian white noise and interference noise, which can affect the detection accuracy.

**Figure 9:**
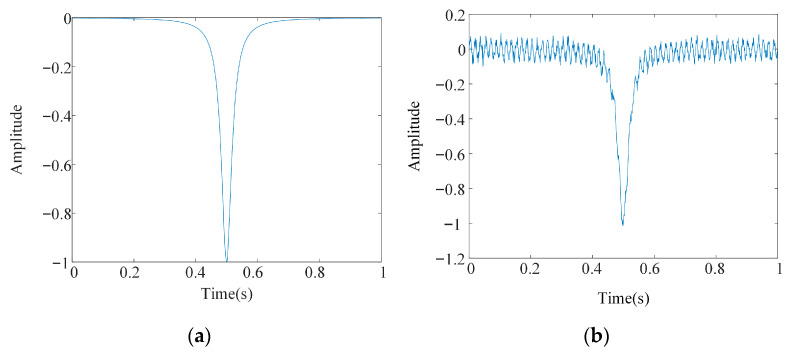
Running time of different denoising methods. (**a**) TDLAS second harmonic signal time-domain plot. (**b**) Noisy TDLAS second harmonic signal.

Under the conditions of a temperature of 296 K and a pressure of 1 atm, the second derivative of the signal is calculated using the five-point difference formula to simulate the second harmonic signal, with its discrete form as follows:

(33) $$H_{2}(\nu_{i})=\frac{\alpha (\nu_{i+2})-30\alpha (\nu_{i+1})+25\alpha (\nu_{i})-18\alpha (\nu_{i-1})+\alpha (\nu_{i-2})}{14{\left(\Delta \nu \right)}^{2}}$$

where α(ν) represents the discrete input signal, Δν represents the step size between frequency points, $H_{2}(\nu )$ represents the output second harmonic signal, and $\nu_{i}$ is the i−th sampling point. A trapezoidal wave is used as the scanning signal, a sine wave as the modulation signal, and Gaussian white noise and interference noise are added. The number of sampling points is set to 1000, generating noise-free and noise-containing CO gas second harmonic simulation signals, as shown in Figure 9.

The ICFO-SVMD-improved wavelet threshold method proposed in this paper is used to denoise the noisy second harmonic signals, and comparisons are made with the traditional wavelet threshold, VMD, and SVMD methods. The signal-to-noise ratio (SNR) and root mean square error (RMSE) results of the denoised signals are recorded in Table 4.

To intuitively evaluate the denoising performance of different methods, the signal-to-noise ratio (SNR) and root mean square error (RMSE) are employed as performance indices for comparative analysis. The SNR reflects the relative strength between the signal and the noise, whereas the RMSE measures the deviation between the denoised signal and the original signal. In general, a smaller RMSE (closer to zero) and a higher SNR indicate better denoising performance. The corresponding formulas are given as follows:

(34) $$R_{\mathrm{SN}}=10\mathrm{lg}\frac{\sum_{i=1}^{N}x^{2}(i)}{\sum_{i=1}^{N}{\left[x(i)-\hat{x}(i)\right]}^{2}}$$

where x(i) represents the original signal value of the i−th sample, $\hat{x}(i)$ represents the estimated value of the i−th sample, N is the total number of samples, $\sum_{i=1}^{N}x^{2}(i)$ is the total energy of the original signal, and $\sum_{i=1}^{N}{\left[x(i)-\hat{x}(i)\right]}^{2}$ is the total energy of the estimation error.

(35) $$R_{\mathrm{MSE}}=\sqrt{\frac{1}{N}\sum_{i=1}^{N}{\left[x(i)-\hat{x}(i)\right]}^{2}}$$

where x(i) represents the true value of the i−th sample, $\hat{x}(i)$ represents the predicted value of the i−th sample, N is the total number of samples, and ${\left[x(i)-\hat{x}(i)\right]}^{2}$ is the squared error of a single sample.

The SNR and RMSE values of each method under different noise levels are summarized in Table 4. The data in Table 4 present the SNR and RMSE indices of various denoising methods at different noise intensities. Under all noise conditions, the proposed method achieves the highest SNR and the lowest RMSE, demonstrating its superior denoising performance.

In summary, the time-domain waveform comparisons in Figure 8 visually confirm the denoising advantage of the proposed method, while the comprehensive quantitative indices in Table 4 provide rigorous evidence from a numerical perspective. The two sets of results are highly consistent and jointly verify the superior performance and reliability of the proposed joint denoising method in terms of signal fidelity and noise suppression.

**Figure 10:**
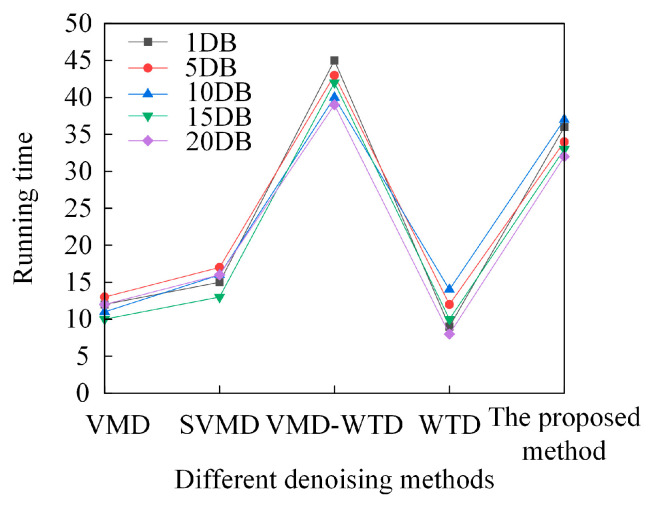
Running time of different denoising methods.

As shown in Figure 10, when dealing with simulation signals under different signal-to-noise ratio (SNR) conditions, the running time of traditional denoising methods is concentrated between 10 and 20 s, while the VMD-WTD-based method reaches 30–50 s. In contrast, the running time of the proposed denoising method remains stable in the range of 30–40 s. The analysis shows that although the proposed method incurs a slightly higher computational cost compared to traditional methods, the increase is within an acceptable range and does not significantly affect its practical usability. Compared to existing methods, the proposed method demonstrates superior denoising quality, achieving a better balance between retaining signal features and suppressing noise. Additionally, the method exhibits good stability: its running time fluctuates minimally under different SNR conditions, indicating strong environmental adaptability, making it suitable for diverse signal processing scenarios in practical engineering applications.

### 3.2. Validation Using Measured Rolling Bearing Data

**Figure 11:**
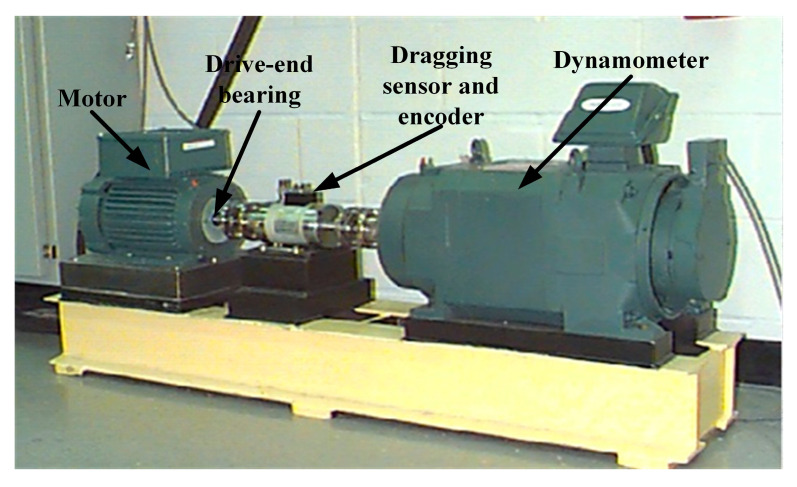
Rolling bearing fault simulation test bench.

To further verify the applicability and effectiveness of the proposed ICFO–SVMD–improved wavelet threshold joint denoising method for practical engineering vibration signals, this section describes the experiments conducted on the publicly available rolling bearing vibration dataset released by Case Western Reserve University (CWRU) [52]. The test bench used for data acquisition is shown in Figure 11. This dataset is widely used as a benchmark for fault diagnosis and denoising algorithm performance evaluation. The test object is a 6205-2RS deep-groove ball bearing (SKF Group, Gothenburg, Sweden), whose main geometric parameters are listed in Table 5. To simulate the noise interference under actual operating conditions and enhance the rigor of the denoising validation, Gaussian white noises of −2 dB, 2 dB, −5 dB, and 5 dB were added to the original measurement signals.

**Figure 12:**
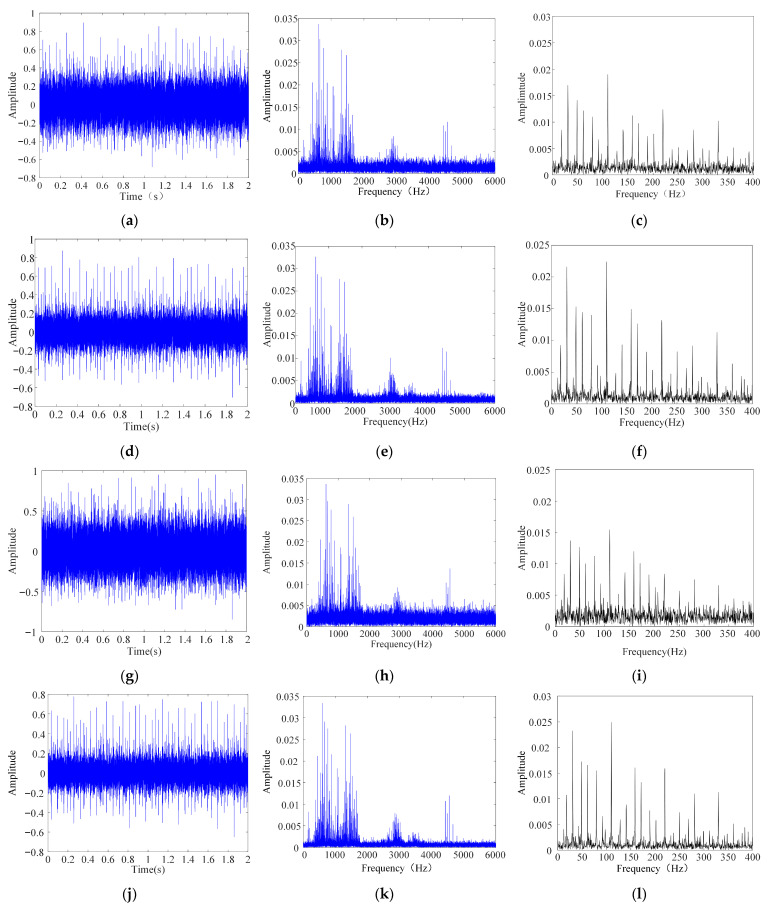
Time and frequency-domain plots of the bearing vibration signal. (**a**) Time-domain graph of bearing signal with −2 dB noise added. (**b**) Frequency-domain graph of bearing signal with −2 dB noise added. (**c**) Envelope spectrum plot of the bearing signal with −2 dB noise added. (**d**) Time-domain graph of bearing signal with 2 dB noise added. (**e**) Frequency-domain graph of bearing signal with 2 dB noise added. (**f**) Envelope spectrum plot of the bearing signal with 2 dB noise added. (**g**) Time-domain graph of bearing signal with −5 dB noise added. (**h**) Frequency-domain graph of bearing signal with −5 dB noise added. (**i**) Envelope spectrum plot of the bearing signal with −5 dB noise added. (**j**) Time-domain graph of bearing signal with 5 dB noise added. (**k**) Frequency-domain graph of bearing signal with 5 dB noise added. (**l**) Envelope spectrum plot of the bearing signal with 5 dB noise added.

Table 5 summarizes the principal geometric parameters of the bearing, including the inner and outer race diameters, rolling element diameter, and characteristic fault frequencies. Among them, the characteristic frequency of the outer race fault is approximately 103.4 Hz. To ensure sufficient time–frequency resolution in the subsequent analysis, the sampling frequency is set to 12,800 Hz with about 25,600 sample points, which can fully cover the operating cycles of typical rotating machinery and their fault response characteristics. The time-domain waveform, spectrum, and envelope spectrum of the measured signal under the outer race fault condition are shown in Figure 12.

In the proposed method, the selection of the number of modes K and the penalty factor α is critical to the performance. The search ranges for both parameters are determined based on the characteristics of vibration signals and typical industrial noise conditions: the number of modes K is set between 2 and 15 to avoid mode mixing and increased computational complexity caused by a high K, or the loss of fault features due to a low K, thus achieving a balance between decomposition adequacy and computational efficiency. The range for the penalty factor α is set from 1000 to 1500, which effectively balances mode separation and maintaining sufficient frequency resolution, facilitating fault feature extraction.

The measured signal is processed using the proposed ICFO–SVMD–improved wavelet threshold method. Taking the minimum envelope entropy as the fitness function, the improved cordyceps fungus optimization algorithm is employed to jointly optimize the SVMD penalty factor α and the number of modes K, where the search range of K is set to [2, 15] and that of α to [1000, 1500]. The optimal parameter pair [10, 1400] obtained by ICFO is then substituted into SVMD for signal decomposition, yielding a series of intrinsic mode functions (IMFs). According to the Pearson correlation coefficient between each IMF and the original signal, highly correlated modes are retained, whereas the remaining noise-dominated modes are discarded. The effective IMF components are further processed using the improved wavelet thresholding scheme to effectively suppress the noise contribution and obtain the reconstructed denoised signal.

**Figure 13:**
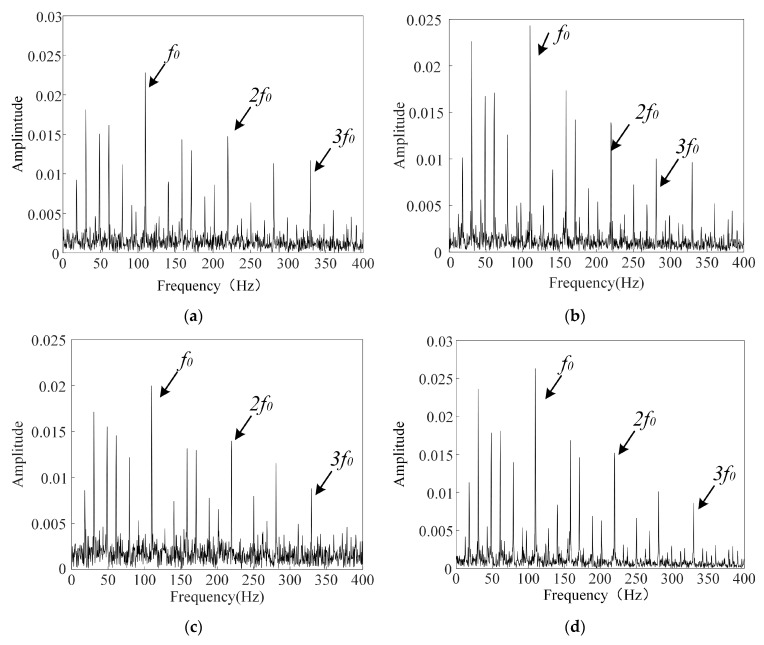
Envelope spectrum of the denoised bearing vibration signal. (**a**) Enveloping spectrum of the bearing signal with −2 dB noise added after denoising. (**b**) Enveloping spectrum of the bearing signal with 2 dB noise added after denoising. (**c**) Enveloping spectrum of the bearing signal with −5 dB noise added after denoising. (**d**) Enveloping spectrum of the bearing signal with 5 dB noise added after denoising.

The envelope spectrum of the rolling bearing signal after denoising is shown in Figure 13. After applying the ICFO–SVMD–improved wavelet threshold denoising method, the noise components in the signal are significantly suppressed, while the key fault features are well preserved. The characteristic frequency associated with the outer race fault is clearly visible in the envelope spectrum with low noise interference. Compared to the original signal, the denoised signal exhibits a higher signal-to-noise ratio and more distinct fault characteristics.

### 3.3. Validation Using Measured Gearbox Data

**Figure 14:**
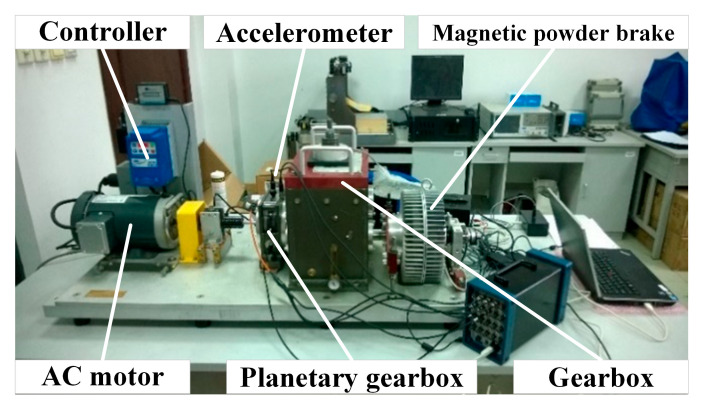
Power transmission simulation laboratory.

To further verify the applicability of the proposed ICFO–SVMD–improved wavelet threshold joint denoising method under practical operating conditions, gearbox fault signals were collected on a dynamic drive simulator (DDS). The test bench mainly consists of a motor, motor controller, planetary gearbox, reduction gearbox, and load, and its structural layout is illustrated in Figure 14. The main parameters of the gearbox used in the experiment are summarized in Table 6, ‘Experimental signal collection parameters’. The experimental data collection parameters are detailed in Table 7.

**Figure 15:**
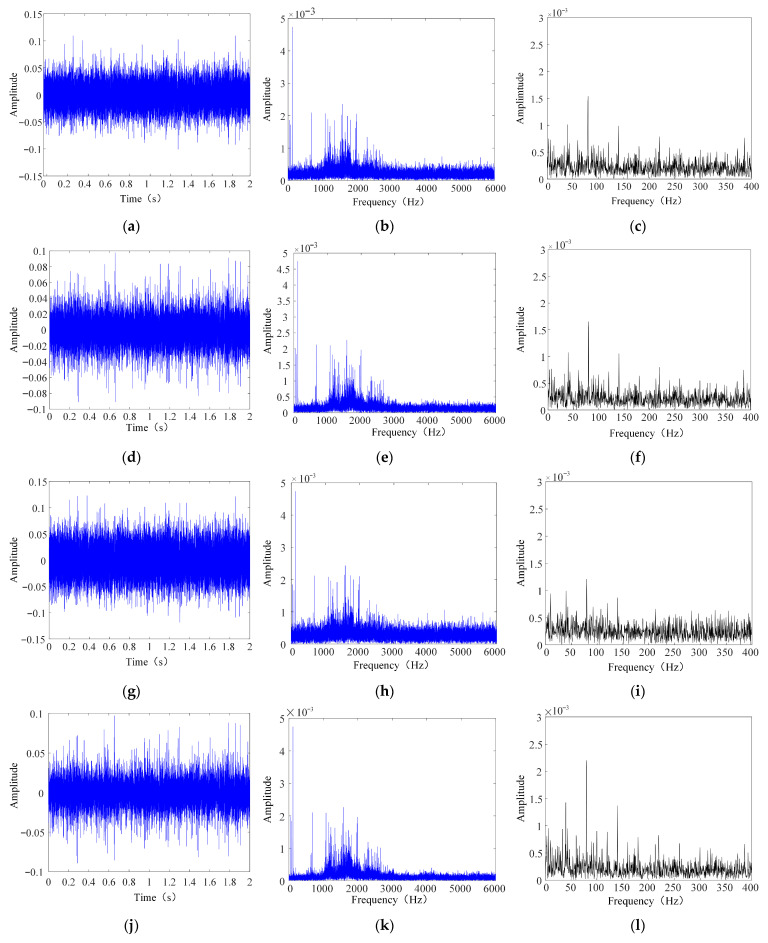
Time- and frequency-domain plots of gearbox vibration signal. (**a**) Time-domain graph of gearbox signal with −2 dB noise added. (**b**) Frequency-domain graph of gearbox signal with −2 dB noise added. (**c**) Envelope spectrum plot of the gearbox signal with −2 dB noise added. (**d**) Time-domain graph of gearbox signal with 2 dB noise added. (**e**) Frequency-domain graph of gearing signal with 2 dB noise added. (**f**) Envelope spectrum plot of the gearing signal with 2 dB noise added. (**g**) Time-domain graph of gearing signal with −5 dB noise added. (**h**) Frequency-domain graph of gearing signal with −5 dB noise added. (**i**) Envelope spectrum plot of the gearing signal with −5 dB noise added. (**j**) Time-domain graph of gearing signal with 5 dB noise added. (**k**) Frequency-domain graph of gearing signal with 5 dB noise added. (**l**) Envelope spectrum plot of the gearing signal with 5 dB noise added.

With regard to fault configuration, a sun gear tooth fracture was introduced, and the system was operated at a constant rotational speed under no-load conditions. The sampling frequency was set to 12,800 Hz, with a total of 25,600 sampling points. Data were collected using a three-axis accelerometer, and the data processing and denoising were performed in MATLAB 2023b. To better approximate the noise environment encountered in industrial applications, Gaussian white noises of −2 dB, 2 dB, −5 dB, and 5 dB were added to the original laboratory test signals. The time-domain waveform, spectrum, and envelope spectrum of the processed test signal are shown in Figure 15.

**Figure 16:**
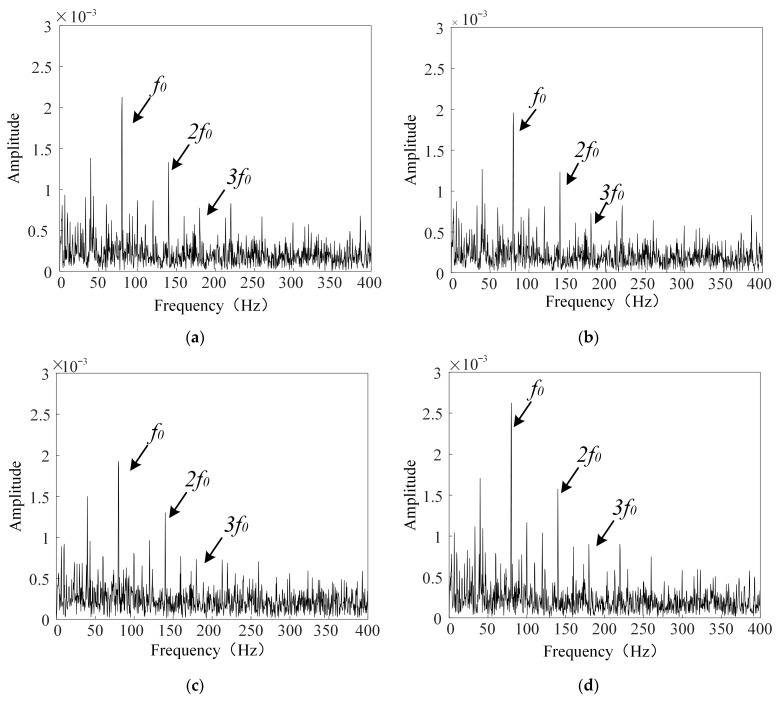
Envelope spectrum of the denoised gearbox vibration signal. (**a**) Enveloping spectrum of the gearbox signal with −2 dB noise added after denoising. (**b**) Enveloping spectrum of the gearbox signal with 2 dB noise added after denoising. (**c**) Enveloping spectrum of the gearbox signal with −5 dB noise added after denoising. (**d**) Enveloping spectrum of the gearbox signal with 5 dB noise added after denoising.

The experimental procedure is identical to that described above, and is therefore not repeated here. The envelope spectrum of the gearbox signal after denoising is shown in Figure 16. By applying the ICFO–SVMD–improved wavelet threshold denoising method, the noise components in the signal are significantly suppressed and the principal fault characteristic frequencies become much clearer. This indicates that the proposed method effectively eliminates noise-induced interference while preserving the key frequency components of the signal, thereby enhancing its interpretability. In particular, a clear envelope spectrum is beneficial for accurately identifying fault patterns in fault diagnosis. These results verify the effectiveness and superiority of the proposed method when applied to practical engineering signals.

Since the experimental data collected are not pure signals, traditional signal-to-noise ratio (SNR) and root mean square error (RMSE) are not suitable for directly evaluating signal quality. To more effectively quantify the denoising performance, in the context of fault feature identification, the residual variance ratio (RVR) and Signal Energy Ratio (SER) become important performance evaluation metrics.

The RVR is used to quantify the proportion of residual noise energy after denoising. The lower the RVR, the more effective signal energy is retained while residual noise is suppressed. Therefore, a lower RVR is directly associated with better feature preservation, helping to extract fault features more completely and improving the accuracy of fault identification in vibration signals.

Similarly, the Signal Energy Ratio (SER) measures the proportion of the original signal energy retained after denoising. A higher SER means that more of the original signal’s energy is preserved after denoising, which is particularly critical for accurately identifying transient and low-amplitude fault features. In strong noise environments, weak fault features can easily be masked by background noise, and enhanced energy retention helps improve the reliability of fault diagnosis, especially under high-noise conditions.

Therefore, both the RVR and SER play a key role in ensuring that the denoising method does not excessively smooth or distort fault features, thus enhancing the robustness and credibility of fault diagnosis in practical engineering applications.

(36) $$RVR=\frac{\sigma_{noise}^{2}}{\sigma_{signal}^{2}}$$

where $\sigma_{noise}^{2}$ is the noise variance and $\sigma_{signal}^{2}$ is the signal variance.

(37) $$SER=\frac{\sum_{n}|s(n){|}^{2}}{\sum_{n}|\eta (n){|}^{2}}$$

where s(n) is the signal sequence and η(n) is the noise sequence.

To further benchmark the superiority of the proposed denoising method, typical approaches such as VMD, SVMD, SVMD–WTD, and VMD–WTD are selected for comparative analysis, and the corresponding results are summarized in Table 8 and Table 9. The experimental data show that, compared with the other methods, the signal processed by the proposed algorithm exhibits a smoother waveform and significantly reduced noise components, making it particularly suitable for denoising non-stationary and nonlinear signals.

### 3.4. Discussion on Practical Industrial Applications

The proposed ICFO–SVMD–WTD joint denoising method can be incorporated as a front-end signal preprocessing module in rotating machinery condition monitoring systems to enhance the distinguishability of fault features under strong background noise and non-stationary operating conditions, thereby providing more reliable inputs for subsequent feature extraction and fault diagnosis. It can be applied to online monitoring rolling bearings and gearboxes in equipment such as motors, pumps, and fans, early warning of weak incipient faults in complex transmission chains, and vibration-signal quality improvement in noisy industrial shop floor environments. Since the proposed method demonstrates a higher post-denoising SNR and better feature fidelity on both simulated signals and measured bearing and gearbox datasets, it has the potential to serve as a general processing component for improving data usability and diagnostic stability in industrial scenarios.

In practical engineering applications, the method can be integrated as follows: first, vibration signals are acquired by an accelerometer and segmented into analysis windows; and second, ICFO is employed to adaptively optimize the SVMD parameters K and α, followed by mode decomposition and correlation-based selection of informative modes; then, the selected modes are denoised using the improved wavelet-thresholding scheme with sub-band SNR modulation and are reconstructed to obtain the cleaned signal; finally, the denoised signal is fed into the existing diagnosis pipeline to achieve more robust fault identification.

## 4. Conclusions

In this paper, a joint vibration–signal denoising method based on an improved cordyceps fungus optimization algorithm, successive variational mode decomposition, and improved wavelet threshold is proposed to address the trade-off between noise suppression and signal fidelity when dealing with non-stationary, multi-component vibration signals. The main work and conclusions are summarized below.

The proposed framework integrates ICFO-optimized SVMD with an improved wavelet thresholding scheme. ICFO is used to adaptively optimize the key SVMD parameters, namely the number of modes K and the penalty factor α, thereby improving decomposition accuracy. Meanwhile, the wavelet threshold is modulated by the sub-band signal-to-noise ratio (sub-band SNR), which strengthens noise suppression while better preserving fine structural details.

The simulation and experimental results consistently verify the superiority of the proposed method under different noise levels. For both the simulated and measured signals, the proposed approach achieves a higher SNR and lower RMSE than conventional methods, effectively suppressing noise while retaining key fault features; for measured signals, it also yields the lowest residual variance ratio (RVR) and the highest Signal Energy Ratio (SER), indicating strong robustness in high-noise environments.

Despite these advantages, the technique still has several limitations. The ICFO-based parameter search introduces additional computational cost, and its performance may depend on the preset search ranges and stopping criteria. In addition, the Pearson-correlation-based mode selection and the wavelet basis/level choices may influence reconstruction quality under varying operating conditions and non-Gaussian noise. Future work will therefore focus on accelerating optimization, developing more adaptive mode-selection and thresholding strategies for diverse noise types and time-varying speeds/loads, and exploring real-time deployment and tighter integration with downstream fault diagnosis models.

Future research will further extend this work in directions that are more aligned with practical engineering applications by validating the proposed ICFO-SVMD-IWTD framework on more real-world vibration datasets covering different rotating machines and operating conditions, assessing its generalization capability beyond the CWRU bearing data and the laboratory gearbox signals.

## Figures and Tables

**Table 1:** Test functions.

Function	Function Name	Search Range	Theoretical Optimum
F1	Sphere	[−100, 100] ^n^	0
F2	Schwefel 2.22	[−10, 10] ^n^	0
F3	Schwefel 1.2	[−100, 100] ^n^	0
F4	Schwefel 2.21	[−100, 100] ^n^	0
F5	Quartic	[−1.28, 1.28] ^n^	0
F6	Schwefel	[−500, 500] ^n^	−12,569.5
F7	Ackley	[−32, 32] ^n^	0
F8	Griewank	[−600, 600] ^n^	0
F9	Penalized Levy Function	[−50, 50] ^n^	0
F10	Shekel 5	[0, 10] ^n^	−10
F11	Shekel 7	[0, 10] ^n^	−10
F12	Shekel 10	[0, 10] ^n^	−10

**Note:** n represents the number of independent variables.

**Table 2:** Test function run results.

Function	Algorithm	Avg	Std	Function	Algorithm	Avg	Std	Function	Algorithm	Avg	Std
F1	ICFO	3.00 × 10^−52^	3.93 × 10^−52^	F2	ICFO	5.17 × 10^−32^	4.10 × 10^−32^	F3	ICFO	3.59 × 10^−47^	4.51 × 10^−47^
	CFO	4.16 × 10^−50^	5.87 × 10^−50^		CFO	3.44 × 10^−29^	4.47 × 10^−29^		CFO	6.62 × 10^−45^	6.33 × 10^−45^
	GWO	6.39 × 10^−24^	1.73 × 10^−24^		GWO	1.80 × 10^−12^	2.37 × 10^−12^		GWO	5.78 × 10^−15^	6.51 × 10^−15^
	WOA	2.25 × 10^−31^	2.74 × 10^−31^		WOA	5.55 × 10^−19^	4.47 × 10^−19^		WOA	0.0016	0.0023
	BKA	3.11 × 10^−5^	1.05 × 10^−5^		BKA	0.0116	0.0071		BKA	0.0514	0.0534
F4	ICFO	1.61 × 10^−24^	2.16 × 10^−24^	F5	ICFO	0.0037	0.0014	F6	ICFO	−8411.333	403.7899
	CFO	3.65 × 10^−23^	2.78 × 10^−23^		CFO	0.0142	0.0051		CFO	−7891.829	174.7308
	GWO	1.92 × 10^−9^	1.18 × 10^−9^		GWO	0.0178	0.0062		GWO	−5327.898	629.2535
	WOA	0.0582	0.0749		WOA	0.0179	0.0191		WOA	−7226.936	2343.4520
	BKA	0.0936	0.0041		BKA	43.3795	5.6841		BKA	−6775.798	105.0250
F7	ICFO	1.23 × 10^−12^	1.70 × 10^−12^	F8	ICFO	0	0	F9	ICFO	9.11 × 10^−8^	6.51 × 10^−8^
	CFO	1.69 × 10^−10^	2.06 × 10^−10^		CFO	0.0105	0.0149		CFO	0.0523	0.0381
	GWO	0.0240	0.0060		GWO	0.2078	0.0093		GWO	0.8231	0.8567
	WOA	2.42 × 10^−11^	3.24 × 10^−11^		WOA	3.33 × 10^−16^	1.57 × 10^−16^		WOA	0.0787	0.0782
	BKA	19.9623	0.0010		BKA	327.9205	60.9416		BKA	214,936,344	17,839,308
F10	ICFO	−10.1531	3.54 × 10^−13^	F11	ICFO	−10.4029	1.32 × 10^−12^	F12	ICFO	−10.5364	2.97 × 10^−12^
	CFO	−6.3918	5.3194		CFO	−3.7243	5.02 × 10^−12^		CFO	−2.4245	0.0040
	GWO	−7.5992	3.5980		GWO	−10.3968	0.0072		GWO	−10.5267	0.0058
	WOA	−7.4247	3.3749		WOA	−5.0544	0.0085		WOA	−7.3555	3.1520
	BKA	−6.0941	1.4082		BKA	−7.7294	3.7360		BKA	−7.8263	3.8286

**Table 3:** Correlation coefficients of each IMF component.

IMF	1	2	3
r	0.9998	0.0005	7.47 × 10^−7^

**Table 4:** Evaluation indicators for noise reduction effect.

Signal	Evaluation Index	SNRI = $R_{\mathrm{SNoise}}-R_{\mathrm{SNRAfter}}$					RMSE				
	Original SNR (dB)	1	5	10	15	20	1	5	10	15	20
Signal 1	Proposed Method	12.7285	12.5566	12.6981	13.7067	14.5293	0.1041	0.0670	0.0371	0.0186	0.0095
	VMD	8.7179	8.8875	8.6538	8.9193	8.6716	0.1652	0.1022	0.0590	0.0322	0.0186
	SVMD	11.6289	11.8906	11.6595	11.7761	11.6269	0.1181	0.0723	0.0590	0.0232	0.0133
	VMD-WTD	10.6235	10.7325	10.5447	10.2539	10.1227	0.1326	0.0826	0.0475	0.0276	0.0159
	CFO-WTD	10.4661	11.4562	8.6821	7.8251	7.7625	0.1351	0.0760	0.0588	0.0365	0.0207
	WTD	11.9433	11.6615	10.7829	9.6686	8.6639	0.1139	0.0743	0.0462	0.0295	0.0186
Signal 2	Proposed Method	13.471	13.820	14.476	15.261	15.733	0.0340	0.0213	0.0124	0.0082	0.0065
	VMD	7.489	6.622	4.512	2.022	0.824	0.0673	0.049	0.038	0.0373	0.0365
	SVMD	7.0848	6.0312	4.6655	3.0416	1.9228	0.0688	0.0545	0.0474	0.0434	0.0315
	VMD-WTD	9.3051	7.1248	3.9554	1.5808	0.9425	0.0545	0.0474	0.0434	0.0401	0.0358
	CFO-WTD	11.2678	12.782	11.7867	11.1627	9.7931	0.0457	0.0237	0.0163	0.0131	0.0128
	WTD	11.9049	11.5594	9.5493	6.4392	3.66	0.0420	0.0283	0.0223	0.0220	0.0259

**Note: *R*_SNoise_** is the signal-to-noise ratio before noise reduction, and $R_{\mathrm{SNRAfter}}$ is the signal-to-noise ratio after noise reduction.

**Table 5:** Basic parameters of the bearing.

Name/Unit	Parameters/Unit	Values
Bearing	Outer diameter/mm	25
	Inner diameter/mm	52
	Width/mm	15
Bearing fault	Rotation frequency/Hz	28.68
	Fault frequency/Hz	103.4
	Fault diameter/mm	0.5334

**Table 6:** Experimental signal collection parameters.

Parameter Name	Set Value
Sampling Frequency	12,800 Hz
Sampling Duration	60 s
Signal Length	768,000 points (per channel)
Sensor Installation	Mounted on the outer ring of the rotating shaft
Collection Channels	X, Y, Z axes for acceleration
Data Format	Time-domain format (.txt/.mat)

**Table 7:** Planetary gearbox parameters.

Component	Number of Gear Teeth	
	Stage 1	Stage 2
Sun gear	20	28
Planet gear	40 × 3	36 × 4
Ring gear	100	100

**Table 8:** Quantitative evaluation of denoising effects of different algorithms (CWRU Dataset).

Operating Conditions	The Proposed Denoising Method	RVR	SER
Operating condition 1	VMD	0.1176	0.4869
	SVMD	0.1008	0.5728
	SVMD-WTD	0.1375	0.5739
	VMD-WTD	0.1158	0.5373
	ICFO-SVMD-WTD	0.0854	0.9221
Operating condition 2	VMD	0.1208	0.4772
	SVMD	0.1143	0.5507
	SVMD-WTD	0.1481	0.5843
	VMD-WTD	0.1358	0.5386
	ICFO-SVMD-WTD	0.0874	0.9317

**Table 9:** Quantitative evaluation of denoising effects of different algorithms (DDS Dataset).

Operating Conditions	The proposed Denoising Method	RVR	SER
Operating condition 1	VMD	0.1266	0.4269
	SVMD	0.1031	0.5208
	SVMD-WTD	0.1452	0.5610
	VMD-WTD	0.1262	0.5058
	ICFO-SVMD-WTD	0.0864	0.8907
Operating condition 2	VMD	0.1253	0.4392
	SVMD	0.1037	0.5083
	SVMD-WTD	0.1578	0.5781
	VMD-WTD	0.1308	0.5213
	ICFO-SVMD-WTD	0.0870	0.8547

## Data Availability

Data are contained within the article.
